# MicroED structure of the NaK ion channel reveals a Na^+^ partition process into the selectivity filter

**DOI:** 10.1038/s42003-018-0040-8

**Published:** 2018-05-03

**Authors:** Shian Liu, Tamir Gonen

**Affiliations:** 1 Janelia Research Campus, Howard Hughes Medical Institute, 19700 Helix Drive, Ashburn, VA, 20147 USA; 2 Howard Hughes Medical Institute, University of California, Los Angeles, CA 90095 USA; 3 Departments of Physiology and Biological Chemistry, David Geffen School of Medicine, University of California, Los Angeles, CA 90095 USA

## Abstract

Sodium (Na^+^) is a ubiquitous and important inorganic salt mediating many critical biological processes such as neuronal excitation, signaling, and facilitation of various transporters. The hydration states of Na^+^ are proposed to play critical roles in determining the conductance and the selectivity of Na^+^ channels, yet they are rarely captured by conventional structural biology means. Here we use the emerging cryo-electron microscopy (cryoEM) method micro-electron diffraction (MicroED) to study the structure of a prototypical tetrameric Na^+^-conducting channel, NaK, to 2.5 Å resolution from nano-crystals. Two new conformations at the external site of NaK are identified, allowing us to visualize a partially hydrated Na^+^ ion at the entrance of the channel pore. A process of dilation coupled with Na^+^ movement is identified leading to valuable insights into the mechanism of ion conduction and gating. This study lays the ground work for future studies using MicroED in membrane protein biophysics.

## Abstract (web summary)

Through a MicroED structure of NaK ion channel, Shian Liu and Tamir Gonen identify a process of dilation coupled with Na^+^ movement, providing mechanistic insights into ion conduction and gating. This study lays the ground work for future studies using MicroED in membrane protein biophysics.

## Introduction

Tetrameric cation channels comprise a superfamily of membrane proteins that mediate ion conduction across membranes. Their roles in electrical signaling have been extensively studied in eukaryotic systems^1^ and more recently in bacterial biofilms^2^. These channels have various strategies to conduct or to exclude some of the most abundant cations in nature, Na^+^ and K^+^ ions. For example, the conserved selectivity filter of K^+^ channels (TVGYG) form a narrow pore lined with four oxygen cages, displacing water from an incoming K^+^ ion to allow efficient conduction^3,4^ (Supplementary Fig. 1a). However, as sodium tries to enter, the K^+^ channel selectivity filter either collapses into an inactivated conformation to stop the conduction of Na^+^^5,6^, or the channel conducts significantly slowly because of unfavorable kinetics^7,8^. Several classes of ion channels, such as voltage-gated Na^+^ (Na_V_) channels, contain a relatively shorter but wider filter than K^+^ channels, and they have been proposed to conduct partially hydrated Na^+^ ions ^9,10^ although these states have never been structurally captured.

To investigate how Na^+^ interacts with ion channels, we applied micro-electron diffraction (MicroED) to study a prototypical cyclic nucleotide-gated ion channel, NaK, as a model system. NaK belongs to the tetrameric cation channel family^11^, and electrophysiology studies have shown that it efficiently conducts Na^+^ ions^12^. Previous X-ray crystallographic structures of NaK revealed a unique architecture of its selectivity filter (_63_TVGDGN_68_) with two distinct regions separated by an internal vestibule^11,13^ (Supplementary Fig. 1b). The intracellular side of the filter consists of two ion-binding sites corresponding to sites 3 and 4 of K^+^ channels, while near the extracellular side of the vestibule, a wide external site is formed by four asparagine residues arranged as a ring. X-ray structures of Na_V_ channels indicated that asparagine or glutamate residues may form direct contacts with ions in the filter;^14–16^ however, no electron density for Na^+^ ions was previously identified at NaK’s external site even after the crystals were soaked in high-salt solutions^13^.

MicroED is a recently developed cryo-electron microscopy (cryoEM) method that applies electron diffraction to solve protein structures from nano-crystals^17^, with which several novel structures have been obtained at atomic resolutions^18–20^. The crystals studied by MicroED are a billion times smaller than what is regularly used in X-ray studies^17^. An important advantage of MicroED over X-ray crystallography is that MicroED provides information about the charges in the proteins^21^. Here, we demonstrate the successful application of MicroED to study the membrane protein crystals, namely the NaK ion channel, where two newly identified conformations underlie the ion conduction and gating mechanisms.

## Results

**Fig. 1:**
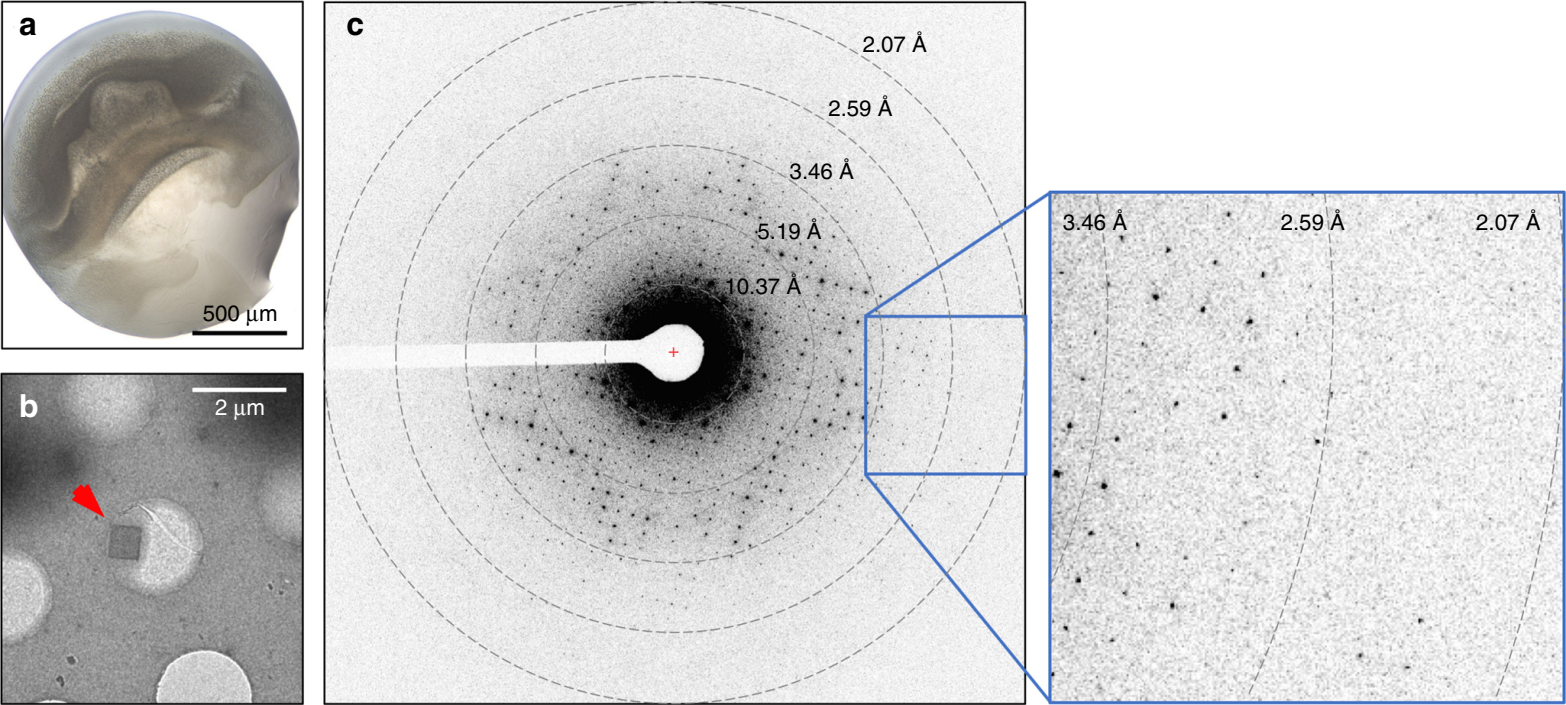
Microscopic analysis of NaK channel nano-crystals. **a**, **b** NaK nano-crystals are imaged under optical and electron microscopes, respectively. Comparing the scales of two images illustrates that the crystals are indistinguishable by optical microscopy (**a**) but clearly resolved under electron microscopy as cubes with sharp edges (**b**, red arrow head). **c** An example of an electron diffraction pattern of a NaK nano-crystal. Data were recorded by continuous rotation showing clear reflections to atomic resolution (~2 Å). Clearly defined reflections at high resolutions are shown in a magnified image on the right

In this study, the first 19 residues of wild-type NaK from *Bacillus cereus* were truncated to create a 10-kDa monomer that assembles as a tetramer forming an open channel construct NaK–∆19^22^, which for simplicity will be referred to as NaK. A protein with this molecular weight is far too small for meaningful structure determination by single-particle cryoEM^23^ but can be studied crystallographically. Therefore, we attempted to crystallize NaK in a 96-well plate in Na^+^ buffers as previously reported^13,22^. Only 0.2 μl of protein was used per condition mixed with an equal volume of the crystallization buffer. Within ~3 days, some drops looked opaque as if filled with protein granular aggregates (Fig. 1a). We speculated that nano-crystals might have formed in these drops, but due to their minute size they were unidentifiable by light microscopy^20^. Indeed, when viewed under the electron microscope, numerous NaK nano-crystals with cubic morphology were found in almost all the drops that contained granular aggregates (Fig. 1b). Such crystallization drops are typically ignored as failed attempts at growing large crystals for X-ray diffraction; however, they are suitable for structure determination by MicroED^24^.

MicroED is an ideal method for studying membrane protein structures from nano-crystals without further crystal growth optimization. First, electrons interact with matter more strongly than X-rays^25^, making it possible to study crystals a billion times smaller in size than what is needed for traditional X-ray crystallographic studies^17,20^. Second, electrons are negatively charged particles, hence they are sensitive to the charges in proteins, suggesting that perhaps one could identify previously unseen ions^21^. The functions of many membrane proteins are coupled to the charge, hence the application of MicroED to unambiguously identify ions within these proteins, as well as protonation states where applicable, can be extremely valuable and important.

**Fig. 2:**
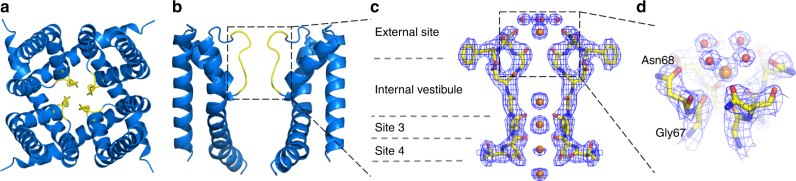
Structural model of the NaK channel determined by MicroED. The top (**a**) and side (**b**) views, respectively, of the overall structure of NaK in a cartoon representation (blue) and the selectivity filter in yellow. (The front and back subunits in **b** are removed for clarity). **c** The selectivity filter region (yellow) in **b** is shown in the stick representation for closer examination, overlaid with 2*F*o–*F*c MicroED density map contoured at 1.5*σ* for molB. Densities inside the selectivity filter include Na^+^ ions and waters shown as orange and red spheres, respectively. **d** The external site bound with a partially hydrated Na^+^ ion in **c** is magnified and shown in a three-dimensional model with the density map

NaK nano-crystals were only ~500 nm in length (Fig. 1b) and readily diffracted to ~2.0 Å resolution by MicroED (Fig. 1c). MicroED data were recorded by continuous rotation as a movie^26^, and the data from 11 crystals were merged to increase the completeness. These crystals had the same unit cell dimensions and symmetry as previously reported (Table 1). The X-ray structure of NaK (PDB accession number 3E89) was used as a search model for molecular replacement, and refinement was performed as described before^27^. The final MicroED NaK model was refined to 2.5 Å resolution having acceptable *R*_work_ and *R*_free_ statistics (21.83% and 26.25%, respectively). The density was of high quality allowing the identification of all amino acids, as well as water molecules and Na^+^ ions (Fig. 2).

**Table 1:** MicroED data collection and refinement statistics

	NaK
*Data collection*	
Space group	I4
Cell dimensions	
* a*, *b*, *c* (Å)	68.07, 68.07, 89.3
*α*, *β*, *γ* (°)	90, 90, 90
Resolution (Å)	22.0–2.5 (2.6–2.5)^a^
*I* / *σI*	4.7 (1.6)
Completeness (%)	81.68 (69.48)
Redundancy	4.9 (3.6)
CC_1/2_ (%)	98.4 (24.3)
*Refinement*	
Resolution (Å)	2.5
No. reflections	5793
*R*_work_/*R*_free_ (%)	21.83 / 26.25
No. atoms	
Protein	1470
Ligand/ion	20
Water	4
*B*-factors	
Protein	41.36
Ligand/ion	41.28
Water	22.78
R.m.s. deviations	
Bond lengths (Å)	0.003
Bond angles (°)	0.47
Ramachandran plot	
Favored (%)	95.63
Allowed (%)	4.37
Outliers (%)	0

^a^ Eleven crystals were merged. Values in parentheses are for highest-resolution shell

**Fig. 3:**
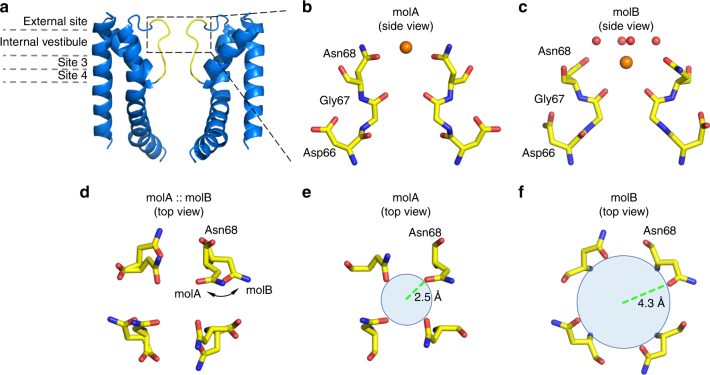
Insertion of partially hydrated Na^+^ into the dilated external site of NaK. **a** Side view of the overall structure (blue) of NaK in a cartoon representation, while the front and back subunits are removed for clarity. Selectivity filter in yellow. **b**, **c** The top half of the filters from molA and molB are shown in stick models, and Na^+^ ions and waters as orange and red spheres, respectively. **d** The top view of the external sites from molA and molB are overlaid to illustrate the conformational change of Asn68 dilating the external site to allow Na^+^ into the pore. **e**, **f** The top view of molA and molB, respectively, illustrating the large conformational change in Asn68 and the resulting change in diameter. As the conformation of Asn68 changes between molA and molB, the radius of the external site almost doubles. Green dashed lines indicate the radii of the circles

As with other tetrameric ion channels, the ion pathway of NaK is located at the four-fold axis of the channel (Fig. 2a). Each NaK monomer is folded into two transmembrane helices (TMs) and one loop that contains the selectivity filter (Fig. 2b). Each unit cell contained two NaK tetramers (referred to as molA and molB) packed in a head-to-head fashion consistent with previous studies^22^. Superposition of NaK determined by MicroED with NaK determined previously by X-ray crystallography (referred to as molC)^22^ indicated a near identical match of the overall architecture with a root mean square deviation (r.m.s.d.) of 0.6 Å (Supplementary Fig. 2). Despite these similarities, there are unique differences present in the MicroED structure. For example, close examination of the selectivity filter revealed several densities for ions and waters at the external site that were not found in the previous studies (Figs. 2, 3 and Supplementary Fig. 1). Moreover, density corresponding to a Na^+^ ion appeared at site 4 at the center of the oxygen cage in NaK instead of at the oxygen plane as seen in the X-ray structures (Supplementary Fig. 1b). While the previous X-ray study identified contaminating ions in the filter, careful mass spectroscopy analysis on our preparations did not identify such contamination (Table 2).

**Table 2:** ICP-MS analysis of ion species in the crystallization condition

Element	Result	Element	Result
Lithium	<2 ppm	Indium	<2 ppm
Beryllium	<2 ppm	Tin	<2 ppm
Boron	<20 ppm	Antimony	<2 ppm
Sodium	1508 ppm	Tellurium	<2 ppm
Magnesium	5 ppm	Cesium	<2 ppm
Aluminum	<20 ppm	Barium	<2 ppm
Phosphorus	<20 ppm	Lanthanum	<2 ppm
Potassium	<20 ppm	Cerium	<2 ppm
Calcium	N/A	Praseodymium	<2 ppm
Scandium	<2 ppm	Neodymium	<2 ppm
Titanium	<2 ppm	Samarium	<2 ppm
Vanadium	<2 ppm	Europium	<2 ppm
Chromium	<2 ppm	Gadolinium	<2 ppm
Manganese	<2 ppm	Terbium	<2 ppm
Cobalt	<2 ppm	Dysprosium	<2 ppm
Nickel	<2 ppm	Holmium	<2 ppm
Copper	15 ppm	Erbium	<2 ppm
Zinc	<20 ppm	Thulium	<2 ppm
Gallium	<2 ppm	Ytterbium	<2 ppm
Arsenic	<2 ppm	Lutetium	<2 ppm
Selenium	<2 ppm	Hafnium	<2 ppm
Rubidium	<2 ppm	Tantalum	<2 ppm
Strontium	<2 ppm	Tungsten	<2 ppm
Yttrium	<2 ppm	Rhenium	<2 ppm
Zirconium	2 ppm	Iridium	<2 ppm
Niobium	<2 ppm	Platinum	<2 ppm
Molybdenum	<2 ppm	Mercury	<2 ppm
Ruthenium	<2 ppm	Thallium	<2 ppm
Rhodium	<2 ppm	Lead	<2 ppm
Palladium	<2 ppm	Bismuth	<2 ppm
Silver	<2 ppm	Thorium	<2 ppm
Cadmium	<2 ppm	Uranium	<2 ppm

**Fig. 4:**
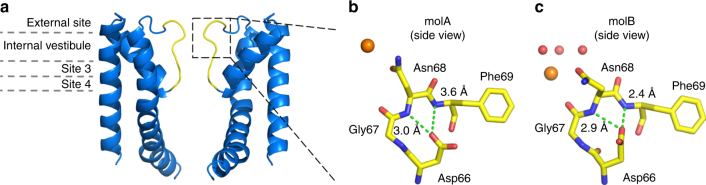
Hydrogen bonding in NaK structures to stabilize Asn68. **a** Side view of the overall structure (blue) of NaK in a cartoon representation, while the front and back subunits are removed for clarity. **b**, **c** The external sites from molA and molB monomers are shown in stick models, respectively. The hydrogen bonds and corresponding distances are indicated in green dashed lines, and Na^+^ ion and waters as orange and red spheres

The two conformations determined here together with the previously known structure of NaK allow us to put together a molecular movie of ion partitioning into this channel (Fig. 3). The starting point of the ion conduction is likely presented by molA. In molA, a Na^+^ ion is bound at the extracellular site in direct contact with Asn68 (Fig. 3b). Four Asn68 residues form a narrow entry point with an approximate radius of 2.5 Å (Fig. 3d, e). Asn68 is held in place by a system of hydrogen bonds involving Phe69, Gly67, and Asp66 (Fig. 4). Next, the rotation of Asn68 in molB dilates the external site like an iris almost doubling its radius from ~2.5 Å to ~4.3 Å (Fig. 3d, f) and in the process pulling the Na^+^ ion deeper into the channel pore. At this state, Asn68 no longer maintains direct interactions with the ion; instead, the coordination is replaced by water molecules that form a bonding network with Asn68, as well as four Gly67 residues directly below the Na^+^ ion. The system of hydrogen bonds that stabilize Asn68 (involving Phe69, Gly67, and Asp66) is shortened to facilitate the rotation of Asn68 to dilate the external site (Fig. 4c). Finally, molC depicts a channel state in which the Na^+^ ion has entered and the external site dilates further to accommodate the water molecules closer to the filter (Supplementary Fig. 1b).

## Discussion

The mechanism described above for Na^+^ conduction through NaK may be applicable to other Na^+^-conducting channels in symmetric assemblies. The extracellular site or the selectivity filter of many Na^+^-conducting channels contain Asn or Glu residues^14,15,28^ (Supplementary Fig. 3 and 4) that may rotate in a concerted manner similarly to NaK to allow the incoming Na^+^ ion to move deeper into the channel. As the partially hydrated sodium is conducted through the pore, the ion could be coordinated directly to the key Asn or Glu residues, or the coordination may involve a H-bridge through water^16,29–31^. In eukaryotes, a single polypeptide chain with four pseudo repeats assemble into a voltage-gated Na^+^ channel. Their filters are formed with a ring of four different residues: Asp, Glu, Lys, and Ala (DEKA)^32^. Although the concerted movement is likely absent, Asp and Glu, and to a lesser extent Lys, may still act in synchrony to attract and guide the associated ions into the channel because of their inherent charge properties. However, additional structures are needed to further delineate the ion conduction mechanism through such asymmetric channels.

Two new conformational states of NaK were captured by MicroED to reveal a novel mechanism of gating at the external site. Two NaK channels pack against one another in a single unit cell, but their extracellular sites are more than 10 Å apart (Supplementary Fig. 2b) indicating that crystal packing does not play a role in dictating the positions of the sodium ions that were identified in this study. Previous studies showed mutant NaK (N68D) had a larger open probability than the wild-type channel without compromising the architecture of the filter^33^. Given the differences between Asn and Asp, our structure suggests that the charge state of the outer pore residues may affect their interaction with passing ions and therefore alter the gating properties. Transient receptor potential (TRP) channels are tetrameric ion channels in a protein family where most members allow Na^+^ ions to pass through and have similar architecture of their outer pore residues (Supplementary Fig. 4). Interestingly, either neutralization of Glu/Asp to Gln/Asn or lowering of the external pH was able to alter the gating properties of many TRP channels^34–38^. Therefore, depending on the specific physiological functions that an activated ion channel is to achieve, gating may be carefully calibrated at the outer pore positions through the interaction with passing ions and fine tuning of their hydration state.

The NaK nano-crystals, used here for MicroED, grew out of a sparse matrix set in a 96-well plate with only 0.2 μl of protein sample per drop. Nano-crystals found in drops that appeared like granular aggregates yielded ~2 Å electron diffraction data without any further optimization of crystal growth. We propose that sparsely populated or transient states in proteins may be teased out when nano-crystals are used in MicroED. Previous MicroED studies with peptides illustrated that different crystal packing of the same sample can arise from one crystallization drop. Likewise, while hundreds of structures of the protein lysozyme have been determined to date, an unprecedented packing was found recently from nano-crystals using MicroED^39^. Whether the crystal size is associated with some differences observed between MicroED and X-ray structures will require further testing, but, when coupled with the unique ability of MicroED to identify charges in proteins, this approach could pave a powerful new way to understanding the structural dynamics in membrane proteins, which is currently beyond the means of conventional X-ray crystallography.

## Methods

### Protein expression

The protein expression and purification procedure was reported previously^13,22^ with some modifications. Briefly, the construct corresponding to the first 19-residue deleted (∆19) NaK was cloned into pQE60 vector. Plasmids were transformed into the XL1-Blue *Escherichia coli* competent cells to allow growth in the presence of 100 μg ml^−1^ ampicillin at 37 °C. When the OD_600_ = 0.8, protein expression was induced with 0.4 mM isopropyl β-D-1-thiogalactopyranoside (IPTG) at 25 °C for 20 h. Cells were harvested by spinning at 4000 rpm using a JLA-8.1 rotor (Beckman Coulter). The cell pellet was suspended in 50 mM Tris buffer (pH 8.0), 150 mM NaCl, 1 mM PMSF, 2 μg ml^−1^ DNase, 10 μg ml^−1^ lysozyme, and Pierce Protease Inhibitors. After passing through a Microfluidizer (Microfluidics Corporation) at 15,000 psi, the cell lysate was spun at 42,000 rpm using Ti45 rotor in Optima L-90K Ultracentrifuge (Beckman Coulter) for 1 h. The pelleted membrane was re-suspended in 50 mM Tris buffer (pH 8.0) and 150 mM NaCl. Homogenized membranes were kept at −80 °C before use.

### Purification

Thawed membranes were mixed with *n*-Decyl-β-D-maltoside (DM) to a final concentration of 2%, and the mixture was stirred at room temperature for 2 h. Insoluble materials were discarded after spinning at 42,000 rpm using Ti70 rotor (Beckman Coulter) for 30 min. The supernatant was applied to a Co^2+^ column (Clontech) pre-equilibrated in 50 mM Tris (pH 8.0), 150 mM NaCl, and 0.2% DM. After washing with 15 mM imidazole and eluting with 300 mM imidazole in the same buffer, NaK was mixed with thrombin protease to a final ratio of 0.5 U mg^−1^ protein. The mixture was incubated at 4 °C overnight, and the digestion was stopped by 1 mM phenylmethanesulfonyl fluoride . Cleaved NaK protein was further purified through a Superdex 200 size exclusion column (GE Healthcare).

### Crystallization

Purified NaK was concentrated to 5–10 mg ml^−1^ for crystallization. A condition matrix was designed for a 96-well plate with (±)-2-methyl-2,4-pentanediol (MPD) concentrations varying between 50 and 80% and pH between 6.0 and 8.5. The pH was buffered with 100 mM MES (pH 6.0), 100 mM MES (pH 6.5), 100 mM HEPES (pH 7.0), 100 mM HEPES (pH 7.5), 100 mM Tris (pH 8.0), and 100 mM Tris (pH 8.5). An inductively coupled plasma mass spectrometry (ICP-MS) assay was performed to ensure that there was no contaminating ion associating with NaK during the crystal growth. The hanging drops were set up with 0.2 μl protein mixed with 0.2 μl reservoir solution, and the plate was incubated at room temperature in the Rock Imager (FORMULATRIX). Since NaK crystals can neither be detected using fluorescence for the lack of tryptophan nor using polarized light, we could only rely on white light to monitor the growth of crystals. Tiny NaK crystals appeared within 3–5 days at room temperature.

### MicroED data collection

Cryo-grids were made as reported previously^24^. The grid screening process of NaK was performed using an FEI Technai F20 field-emission TEM as before^17^. Continuous rotation MicroED data were collected^26^ with a TVIPS TemCam-F416 CMOS camera at the rolling-shutter mode as a movie. Each frame in the movie was recorded as the campustage was rotated at 0.19° s^−1^ during 4 s exposures. Image frames were converted to SMV format for subsequent data processing^27^.

### Structure determination

Each MicroED dataset was indexed and integrated in iMOSFLM^40^, and 11 best datasets were scaled and merged in AIMLESS^41^. Due to the orientation preference of NaK crystals on the grid, cell dimension *c* was detected less precisely than the other five parameters. To determine its value, *c* was manually varied by 0.3 Å each time in sftools in CCP4i package. The resulting file was molecular replaced^42^ with PDB entry 3E89, and maximum likelihood structure refinement was performed in phenix.refine using electron-scattering factors^43^ and coot^44^. This process was iterated several times until the lowest *R* values were found. The final corresponding cell dimensions appeared to be consistent with the X-ray structures reported previously. Water molecules were automatically modeled during the refinement in phenix.refine. The refinement statistics are reported in Table 1.

### Data availability

The data that support the findings of this study are available from the corresponding author upon reasonable request. Coordinates and structure factors for NaK have been deposited in the RCSB Protein Data Bank and Electron Microscopy Data Bank under accession codes 6CPV and EMD-7558, respectively.

## Electronic supplementary material

Supplementary Information
