# Supplementary material for: MicroED structure of the NaK ion channel reveals a Na+ partition process into the selectivity filter
Source: Commun Biol. 2018 May 3;1:38. doi: 10.1038/s42003-018-0040-8 (PMC6112790; doi:10.1038/s42003-018-0040-8)
Supplement: Supplementary file 1 — Supplementary Information [file 42003_2018_40_MOESM1_ESM.pdf]

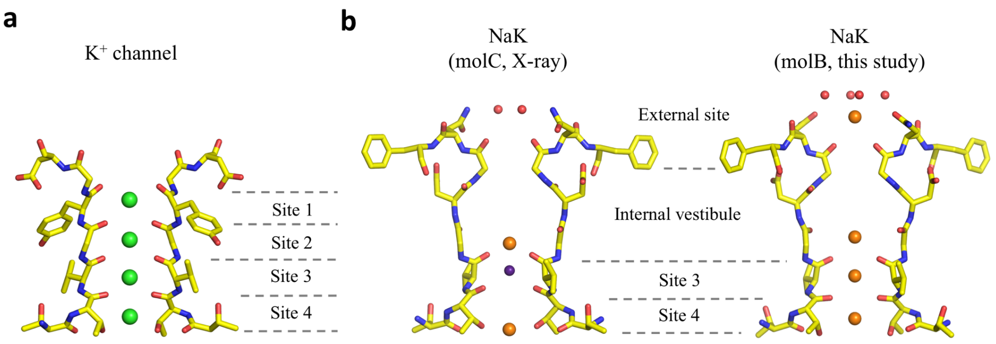

Supplementary Figure 1. Comparison of the filters of K^+ channel and the NaK channel. **a**, The selectivity filter of a K^+ channel (left, PDB accession number 1K4C) with the K^+ binding sites shown in green spheres. **b**, The Na^+ bound NaK manifests differences at the ion binding sites between the X-ray (left, PDB accession number 3E83) and MicroED (right) structures. The Na^+ ions, putative cesium ions and water molecules are presented as orange, purple and red spheres, respectively.

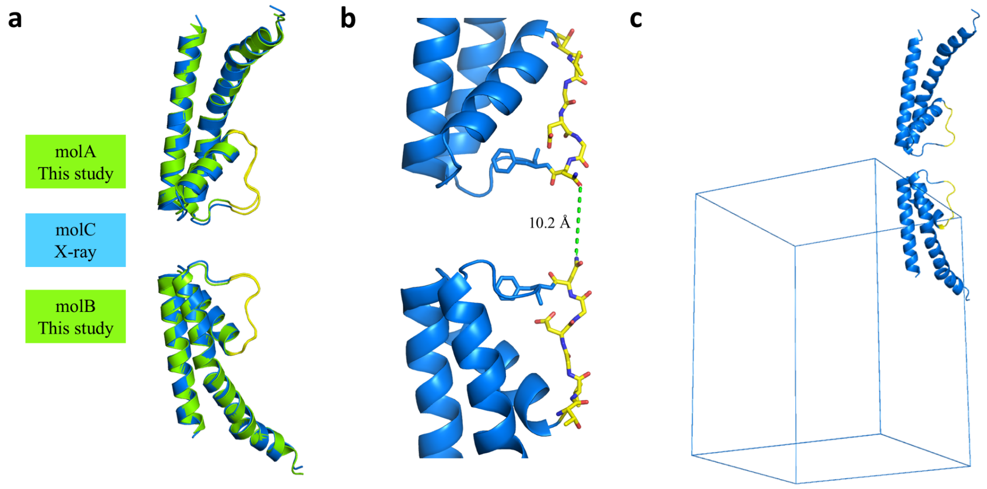

Supplementary Figure 2. Cartoon representation of the overall structure of NaK determined by MicroED (blue) and its selectivity filter (yellow). **a,** Superimposition of the MicroED structure with the X-ray structure (green, PDB: 3E89) is shown. Two opposite monomers found in one asymmetric unit. **b**, Zoom-in images of two opposing monomers to illustrate the absence of contacts between two filters. **c**, The asymmetric unit of NaK is shown with a unit cell.

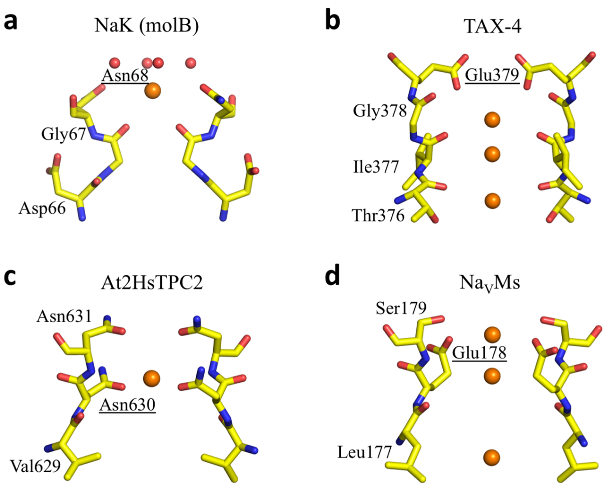

Supplementary Figure 3. Comparison of the external sodium binding site of NaK with the selectivity filters of other Na^+-conducting channels. The opposing subunits of NaK from this study (**a**), a *C. elegans* CNG channel (**b**, PDB accession number 5H3O), an engineered *A. thaliana* channel that mimics the human Na_V two-pore channel (**c**, PDB accession number 5TUA) and Na_VMs from *M. marinus* (**d**, PDB accession number 5BZB) are compared in stick representation. Sodium ions are shown as orange spheres, and key residues are indicated in the structures. Residues that may interact with partially hydrated sodium in a similar manner as Asn68 of NaK are underlined in all structures.

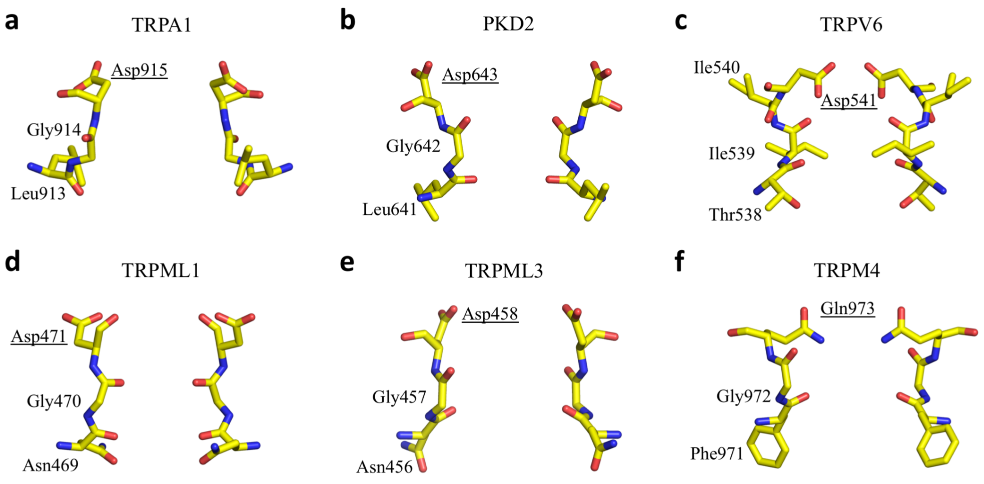

Supplementary Figure 4. Comparison of the filters and outer pore residues among transient receptor potential (TRP) channels. The opposing subunits of TRPA1 (**a**, PDB accession number 3J9P), PKD2 (**b**, PDB accession number 5T4D), TRPV6 (**c**, PDB accession number 5IWP), TRPML1 (**c**, PDB accession number 5WPV), TRPML3 (**c**, PDB accession number 5W3S) and TRPM4 (**d**, PDB accession number 6BCO) are compared in stick representation. Outer pore residues that function in a similar manner as Asn68 of NaK are underlined in all structures.
